# The Lactate/Albumin Ratio: A Valuable Tool for Risk Stratification in Septic Patients Admitted to ICU

**DOI:** 10.3390/ijms18091893

**Published:** 2017-09-02

**Authors:** Michael Lichtenauer, Bernhard Wernly, Bernhard Ohnewein, Marcus Franz, Bjoern Kabisch, Johanna Muessig, Maryna Masyuk, Alexander Lauten, Paul Christian Schulze, Uta C. Hoppe, Malte Kelm, Christian Jung

**Affiliations:** 1 Clinic of Internal Medicine II, Department of Cardiology, Paracelsus Medical University of Salzburg, 5020 Salzburg, Austria; m.lichtenauer@salk.at (M.L.); b.wernly@salk.at (B.W.); b.ohnewein@salk.at (B.O.); u.hoppe@salk.at (U.C.H.); 2 Clinic of Internal Medicine I, Department of Cardiology, Jena University Hospital, 07747 Jena, Germany; Marcus.Franz@med.uni-jena.de (M.F.); Bjoern.Kabisch@med.uni-jena.de (B.K.); Christian.Schulze@med.uni-jena.de (P.C.S.); 3 Division of Cardiology, Pulmonology, and Vascular Medicine, Medical Faculty, University Duesseldorf, Moorenstraße 5, 40225 Dusseldorf, Germany; Johanna.Muessig@med.uni-duesseldorf.de (J.M.); Maryna.Masyuk@med.uni-duesseldorf.de (M.M.); malte.kelm@med.uni-duesseldorf.de (M.K.); 4 Department of Cardiology, Charité—Universitaetsmedizin Berlin, 12203 Berlin, Germany; Alexander.Lauten@charite.de; 5 Deutsches Zentrum für Herz-Kreislauf-Forschung (DZHK), Standort Berlin, 13347 Berlin, Germany

**Keywords:** critically ill patients, lactate, albumin, lactate/albumin ratio, ICU, risk stratification, risk score

## Abstract

The lactate/albumin ratio has been reported to be associated with mortality in pediatric patients with sepsis. We aimed to evaluate the lactate/albumin ratio for its prognostic relevance in a larger collective of critically ill (adult) patients admitted to an intensive care unit (ICU). A total of 348 medical patients admitted to a German ICU for sepsis between 2004 and 2009 were included. Follow-up of patients was performed retrospectively between May 2013 and November 2013. The association of the lactate/albumin ratio (cut-off 0.15) and both in-hospital and post-discharge mortality was investigated. An optimal cut-off was calculated by means of Youden’s index. The lactate/albumin ratio was elevated in non-survivors (*p* < 0.001). Patients with an increased lactate/albumin ratio were of similar age, but clinically in a poorer condition and had more pronounced laboratory signs of multi-organ failure. An increased lactate/albumin ratio was associated with adverse in-hospital mortality. An optimal cut-off of 0.15 was calculated and was associated with adverse long-term outcome even after correction for APACHE2 and SAPS2. We matched 99 patients with a lactate/albumin ratio >0.15 to case-controls with a lactate/albumin ratio <0.15 corrected for APACHE2 scores: The group with a lactate/albumin ratio >0.15 evidenced adverse in-hospital outcome in a paired analysis with a difference of 27% (95%CI 10–43%; *p* < 0.01). Regarding long-term mortality, again, patients in the group with a lactate/albumin ratio >0.15 showed adverse outcomes (*p* < 0.001). An increased lactate/albumin ratio was significantly associated with an adverse outcome in critically ill patients admitted to an ICU, even after correction for confounders. The lactate/albumin ratio might constitute an independent, readily available, and important parameter for risk stratification in the critically ill.

## 1. Introduction

Even to the present day, sepsis, including severe sepsis and septic shock, represents a major health care issue with concerning mortality rates [1,2,3,4,5]. Using certificated death data, sepsis was listed as a cause of death in at least 6% among the causes of in-hospital deaths, which may be estimated as the lower limit [6]. The global trend even shows a rising incidence of sepsis and septic shock [7,8,9] despite generally decreasing mortality rates [10,11], which correlates with the establishment of the Early Goal Directed Therapy (EGDT) guideline [12,13] first published in 2001 [14]. The long-term benefit of EGDT, however, remains still uncertain [15].

The prognosis of critical ill patients is central in clinical routine, especially in consideration of its high relevance in determination of further therapy strategies and increased risk of death even after hospital discharge [16,17]. Focusing on long-term prognosis, data have shown an up to 20% increased risk of death in patients suffering from sepsis as well as an increased risk of developing further sepsis in the future [18].

Multiple predictive scoring systems were developed to objectively evaluate the prognosis and mortality in septic patients; all have shown comparable results [19,20,21]. The scoring system APACHE II reaches a specificity up to 98% but still has a sensitivity of only 85% [22]. We focused on evaluating a new parameter for the prediction of mortality.

Septic patients suffer from low peripheral oxygenation leading to anaerobic glycolysis due to insufficient oxygen delivery, which leads to lactate production [23]. Increased lactate levels were associated with tissue hypoxia since Araki and Zillessen observed this phenomenon when they interrupted oxygen supply to muscles in mammals and birds and noticed that lactic acid was formed in response [24]. In today’s clinical practice, lactate levels are usually used to detect tissue hypoxia; however, increased lactate levels reflect more than just this aspect. The finding of hyperlactatemia in patients with normal tissue perfusion and oxygen delivery additionally suggests that an overstimulation of the Na^+^-K^+^-ATPase leads to an increased lactate production in septic patients similar to hypokalemia [25,26]. It has been shown that activation of the skeletal muscle Na^+^-K^+^-ATPase pump is a relevant factor in lactate production, as it has been observed that lactate is released from muscular tissue in septic shock patients. Several studies have proven that lactate levels are a reliable parameter in diagnosis, therapy evaluation, and prognosis in circulatory shock [27,28,29].

Albumin levels also reflect the severity of inflammation as albumin is a negative acute phase protein. Studies have shown that albumin could serve as an additional parameter for mortality and prognosis [30,31]. Considering that hypoalbuminemia is also a frequent finding in chronic disease, data from predominantly community-acquired sepsis suggested that hypoalbuminemia is related to infection and showed that albumin can serve as an independent risk parameter [32]. Analysis of serum albumin levels after bacteremia and infection serum albumin levels showed an acute decrease as a result of infection, again correlating with poor prognosis [33].

As each of the two parameters independently predicts mortality, a combination of both was meant to further increase the predictive value. We found two studies investigating this field. One focused on pediatric patients and the other on multiple-organ dysfunction syndrome (MODS) and mortality in 54 patients with severe sepsis and septic shock. Both studies show a significant association between the serum lactate/albumin ratio and mortality [34,35]. This current study was designed to evaluate the prognostic value of the serum lactate/albumin ratio in septic patients regarding all-cause mortality in a larger patient cohort (compared to previous studies). 

## 2. Results

### 2.1. Study Population

Compared to survivors, non-survivors were of slightly older age (67.7 vs. 63.7 years, *p* < 0.01). Logically, non-survivors evidenced higher SAPS2 and APACHE2 scores (58.6 vs. 46.4 and 27.8 vs. 24.5, *p* < 0.001 and *p* = 0.001). Non-survivors had more pronounced laboratory signs of multi-organ failure showing higher liver and kidney parameters (see Table 1). As expected, non-survivors also evidenced higher lactate levels (3.8 vs. 2.2 mmol/L, *p* < 0.01) and lower albumin levels (18.0 vs. 20.0 mg/L, *p* = 0.01). An interesting fact was that procalcitonin, leucocyte, and hemoglobin levels did not differ between survivors and non-survivors.

Using Youden’s index, we calculated an ideal cut-off for the lactate/albumin ratio of 0.15 for the prediction of mortality. Patients with an increased lactate/albumin ratio were of similar age but clinically more ill as expressed by both higher SAPS2 (46.4 vs. 58.6, *p* < 0.001) and APACHE2 (24.5 vs. 27.8, *p* = 0.001) scores. Patients with an increased lactate/albumin ratio had more pronounced laboratory signs of organ dysfunction with elevated liver parameters (Table 2). Interestingly, parameters of kidney function did not differ significantly between patients with a lactate/albumin ratio below or above 0.15. However, when dividing our patient cohort into these two subgroups, procalcitonin levels were significantly higher in patients with a lactate/albumin ratio >0.15.

### 2.2. Survival Data

**Figure 1:**
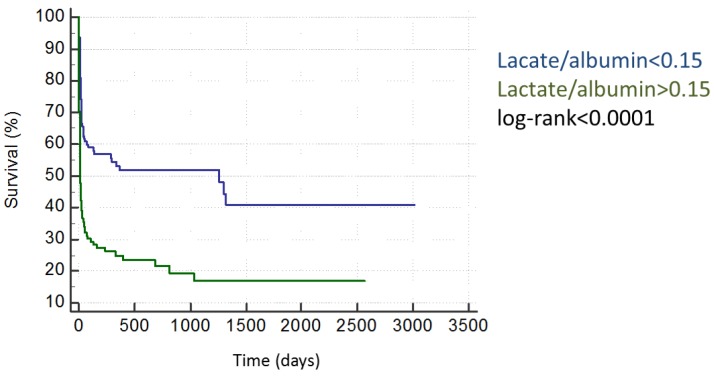
An increased lactate/albumin ratio is associated with adverse long-term outcome.

We performed ROC-analysis (AUC 0.70; 95%CI 0.64–0.76) and evaluated the lactate/albumin ratio for the prediction of in-hospital mortality, and an optimal cut-off was calculated at 0.15 by means of Youden’s index (Table 3). This cut-off was associated with significantly increased in-hospital mortality (54% vs. 18%; *p* < 0.001). A lactate/albumin ratio >0.15 was associated with adverse long-term mortality (Figure 1, HR 2.50 95%CI 1.85–3.37; *p* < 0.001). The association of a lactate/albumin ratio >0.15 with long-term mortality remained significant even after correction for APACHE2 (HR 1.56; 95%CI 1.28–1.91; *p* < 0.001) and SAPS2 (HR 1.47; 95%CI 1.20–1.80; *p* < 0.001) (Table 4).

AUC analysis was performed (AUC 0.755 for albumin alone, AUC 0.804 for lactate alone, and AUC 0.814 for the lactate/albumin ratio), and we found that, in the short-term analysis, albumin alone is inferior (*p* = 0.0001) to the lactate/albumin ratio for the prediction of mortality; lactate analysis was found to be equal (*p* = 0.47 vs. lactate albumin ratio). However, in the long-term analysis, both albumin (AUC 0.717, *p* = 0.043) and lactate levels (AUC 0.719, *p* = 0.035) are inferior to the lactate/albumin ratio (AUC 0.745); the lactate/albumin ratio can add prognostic value for long-term outcome, as it combines a volatile parameter (lactate) that reflects acute disease severity and albumin, which reflects more long-term characteristics such as nutritional status.

### 2.3. Matched-Control Analysis

**Figure 2:**
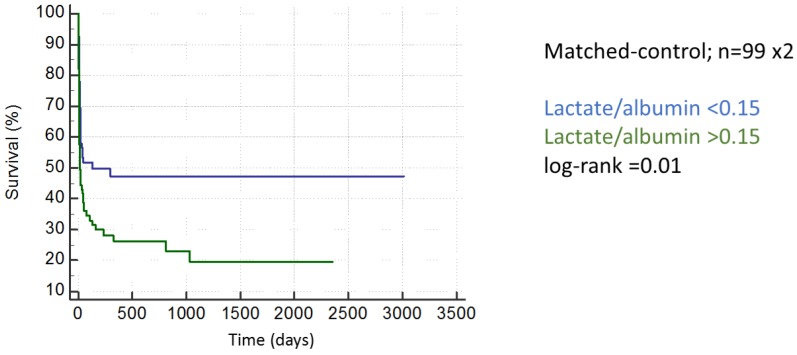
An increased lactate/albumin ratio is associated with long-term mortality in a matched-control analysis.

We matched 99 patients with a lactate/albumin ratio >0.15 to case-controls with a lactate/albumin ratio <0.15 corrected for APACHE2 scores: the group with a lactate/albumin ratio >0.15 evidenced an adverse in-hospital outcome in a paired analysis with the difference being 27% (95%CI 10–43%; *p* < 0.01). Regarding long-term mortality, again, adverse outcomes were observed in patients with a lactate/albumin ratio > 0.15 (Figure 2: log-rank< 0.001).

## 3. Discussion

With the success of initial resuscitation treatments and decreasing death rates for severe sepsis or septic shock, research focus has moved to the later prognosis of this patient population. Our study shows that the lactate/albumin ratio at admission can be used as an independent predictor of mortality in patients with severe sepsis or septic shock. 

To our knowledge, only one small study (*n* = 54, Wang et al.) has evaluated the prognostic significance of the lactate/albumin ratio in septic adults [34]. Most previous studies have investigated the use of single inflammatory markers, such as CRP, procalcitonin, and pro-BNP to predict mortality in critically ill patients. In another clinical study, Choi et al. investigated a lactate/albumin ratio in pediatric patients with sepsis (*n* = 90) [35]. Our current study with 348 enrolled patients represents the largest collective that has been analyzed for the prognostic relevance of the lactate/albumin ratio to date.

Our study suggests that the lactate/albumin ratio is independently associated with the mortality of these patients. An increased lactate/albumin ratio was associated with the development of organ failure. With a cutoff value of 0.15, the lactate/albumin ratio offered good diagnostic sensitivity, specificity, and positive and negative predictive value for multi-organ dysfunction and mortality in these patients. Consistent with previous studies, malnutrition, inflammation, and hypo-perfusion were common in patients with severe sepsis. Hyperlactatemia has been shown to be an independent predictor of mortality in critically ill patients [25,36].

Owing to the mechanisms responsible for lactate accumulation in severe sepsis and septic shock, lactate remains a robust surrogate marker for the development of multi-organ dysfunction in septic patients and poor outcome [25,29]. A recurring theme in previous studies was that inflammatory response plays a crucial mechanistic intermediate between lactate clearance and the development of multi-organ dysfunction. Albumin levels could be a reliable indicator of frailty, high susceptibility to stressors, and unstable homeostasis and were also associated with prognosis after critical illness [36].

As lactate and albumin levels evidence a divergent course as the development of sepsis progresses, a ratio between the two could serve as a novel and maybe better indicator for the prognosis of the patient. Taken together with these markers, the lactate/albumin ratio at admission can be used as a novel predictor to stratify patients according to the severity of disease, even after the patients have been discharged from hospital.

## 4. Methods

### 4.1. Study Subjects

A total of 348 patients that were treated for sepsis or septic shock at an ICU at the University Hospital Jena between January 2004 and December 2009 were enrolled in this study. Inclusion criteria were admission to the medical ICU at University Hospital Jena, and available data on the admission lactate/albumin ratio. The study was approved by the local ethics committee at the University Hospital Jena (#2762-02/10). Standard laboratory values, patient’s medical history, and clinical data were documented. All laboratory values were obtained from the first blood withdrawal once the patient was admitted to our ICU and were analyzed at the Department for Laboratory Medicine at the University Hospital Jena. Follow-up of patients was performed retrospectively between May 2013 and November 2013. Sepsis was defined as systemic inflammatory response syndrome (SIRS) in response to an infection with signs of bacteremia and/or sepsis-induced tissue hypoperfusion or organ dysfunction [37] warranting intensive care treatment. Diagnostic and therapeutic decisions were made by trained physicians in intensive care medicine. The primary endpoint of the study was all-cause mortality. Mortality data were collected by review of medical records in our COPRA patient data management system (COPRA System GmbH, Berlin, Germany) and/or patient contact for mortality analysis of up to 7 years. 

### 4.2. Statistical Analysis

Normally distributed data points are expressed as mean ± standard error of the mean. Differences between independent groups were calculated using ANOVA. Categorical data are expressed as numbers (percentage). A chi-square test was applied to calculate differences between groups. Survival rates were calculated using a chi-square test for analysis of intra-ICU mortality and both univariate and multivariate Cox regression analysis to adjust for confounding factors for long-term mortality. For the multivariate regression model, confounders with a *p*-value <0.10 in the univariate analysis were included, and a backward variable elimination was then performed. Elimination criterion was a *p*-value of more than 0.10. Moreover, a matched case–control analysis was performed by matching 99 patients with a lactate/albumin ratio >0.15 to case-controls evidencing a lactate/albumin ratio <0.15 corrected for APACHE2 scores. ROC analysis was performed, and area under the curve (AUC) was calculated. A *p*-value of <0.05 was considered statistically significant. 

SPSS version 22.0 (Chicago, IL, USA) and MedCaLC24 version 17.4.4 (MedCaLC24 Software, Ostend, Belgium) were used for all statistical analyses.

### 4.3. Calculation of SAPS2 and APACHE score

Initial Simplified Acute Physiology Score II (SAPS2) and Acute Physiology And Chronic Health Evaluation (APACHE) scores were calculated by the treating physician within 24 h after admission as previously reported [38].

## 5. Limitations

There were several limitations to our study. First, as this was a retrospective study, a selection bias was possible. Second, the variables were collected from a single center, making generalization of these results to other institutions difficult. Moreover, we do not have data on albumin replacement, the start of antibiotic treatment, or the total amount of fluids administered. Additional prospective studies with larger populations involving multiple centers are necessary to accurately evaluate the CRP/albumin ratio as a predictor of mortality.

## 6. Conclusions

An increased lactate/albumin ratio correctly identifies patients who were clinically in a poorer condition, suffered from more concomitant diseases, and evidenced more pronounced laboratory signs of multi-organ failure. The lactate/albumin ratio was robustly associated with both in-hospital and post-discharge mortality in our study cohort. As this remained true both after correction for several relevant confounders in a multivariate analysis as well as in matched-control analysis, we believe that the lactate/albumin ratio might constitute an independent risk parameter, with additive value to established and complex scores for risk stratification. The lactate/albumin ratio is very easy to obtain and might therefore be very useful for risk stratification in critically ill patients.

## Figures and Tables

**Table 1:** Laboratory and clinical baseline characteristics; survivors vs. non-survivors.

Clinical Characteristics	Survivors			Non-Survivors			Overall Cohort			*p*-Value
	Mean	SEM		Mean	SEM	Mean	SEM			
age	63.67	14.71		67.67	12.02	64.97	14.00			0.01
BMI (kg/m^2^)	27.06	4.80		27.63	7.83	27.24	5.95			0.47
SAPS2 (pts)	46.43	16.70		58.57	19.37	50.58	18.55			<0.001
APACHE2 (pts)	24.50	7.81		27.84	8.44	25.64	8.17			0.001
	**Median**	**Min.**	**Max.**	**Median**	**Min.**	**Max.**	**Median**	**Min.**	**Max.**	***p*-Value**
lactate (mmol/L)	2.19	0.50	20.58	3.77	0.70	22.90	2.50	0.50	22.90	<0.001
PCT (mmol/L)	8.82	0.08	417.00	7.82	0.07	194.00	8.18	0.07	417.00	0.98
glucose (mmol/L)	9.9	3.9	30.0	10.4	3.8	39.4	10.1	3.8	30.0	0.11
hemoglobin (mmol/L)	6.60	2.50	10.30	6.60	4.80	8.90	6.60	2.5	10.30	0.35
ASAT (μmol/L·s)	0.85	0.20	222.27	1.07	0.20	154.50	0.89	0.20	222.27	0.02
ALAT (μmol/L·s)	0.58	0.08	41.81	0.73	0.10	71.70	0.63	0.08	71.70	0.03
γ-GT (μmol)	1.24	0.10	15.22	1.44	0.14	14.68	1.31	0.10	15.22	0.14
bilirubin (μmol)	17.0	1.8	405.0	19.0	3.0	551.0	18.0	1.8	551.0	0.28
leucocytes (G/L)	14.2	0.1	195.1	14.9	0.2	70.9	14.5	0.1	195.1	0.83
BUN (mg/dL)	12.8	1.9	59.6	19.5	0.5	80.2	14.4	0.5	80.2	<0.001
creatinine (mg/dL)	15.0	30.0	1609.0	212.5	23.0	952.0	175.0	23.0	1609.0	<0.001
sodium (mmol/L)	140	119	154	142	131	151	140	119	154	0.02
potassium (mmol/L)	4.1	2.5	9.3	4.5	3.1	10.8	4.2	2.5	10.8	<0.001
albumin (mg/L)	20.00	10.00	57.00	18.00	10.00	27.00	19.00	10.00	57.00	0.01
lactate/albumin ratio	0.09	0.02	0.15	0.31	0.15	2.00	0.14	0.02	2.00	<0.001

**Table 2:** Laboratory and clinical baseline characteristics; lactate/albumin >0.15 vs. lactate/albumin <0.15.

Clinical Characteristics	Lactate/Albumin <0.15			Lactate/Albumin >0.15			*p*-Value
	Mean	SEM		Mean	SEM		
age	63.70	14.75		66.23	12.69		0.09
BMI (kg/m^2^)	27.33	5.12		27.14	4.82		0.77
SAPS2 (pts)	47.81	18.03		59.01	20.82		<0.001
APACHE2 (pts)	25.07	8.38		27.98	8.24		<0.001
**Laboratory Parameters**	**Median**	**Min.**	**Max.**	**Median**	**Min.**	**Max.**	***p*-Value**
lactate (mmol/L)	1.7	0.5	5.9	5.9	1.8	26.0	<0.001
procalcitonin (mmol/L)	6.5	0.1	161.9	10.1	0.2	188.6	<0.001
glucose (mmol/L)	9.9	5.3	25.3	9.7	3.9	30.0	0.95
hemoglobin (mmol/L)	6.6	2.5	9.3	6.7	4.9	9.8	0.36
ASAT (μmol/L·s)	0.75	0.20	106.95	1.44	0.20	154.50	<0.001
ALAT (μmol/L·s)	0.61	0.13	78.76	0.77	0.10	71.7	<0.001
γ-GT (μmol)	1.29	0.1	15.22	1.3	0.14	20.33	0.92
bilirubin (μmol)	17.0	1.8	390.0	24.0	2.0	551.0	<0.001
leucocytes (G/L)	12.9	0.1	50.5	15.4	0.1	96.8	0.06
BUN (mg/dL)	15.0	0.5	80.2	15.8	1.9	75.4	0.34
creatinine (mg/dL)	174	30	870	192	52	1332	0.34
sodium (mmol/L)	139	124	158	142	126	158	0.02
potassium (mmol/L)	4.2	2.9	7.1	4.5	2.8	12.4	0.01
albumin (mg/L)	21	10	60	17	10	30	<0.001

**Table 3:** An increased lactate/albumin ratio is associated with adverse in-hospital outcome.

Parameter	HR	95%CI	*p*-Value	Non-Survivors Lactat/Albumin >0.15	vs.	Non-Survivors Lactat/Albumin <0.15
lactate/albumin >0.15	4.27	2.42–7.52	<0.001	54%	vs.	18%

**Table 4:** A lactate/albumin ratio >0.15 is associated with long-term mortality after correction for several confounders in a multivariate analysis.

Multivariate Analysis	Univariate			Multivariate		
**Model #1**	**HR**	**95%CI**	***p*-Value**	**HR**	**95%CI**	***p*-Value**
lactate/albumin >0.15	2.5	1.85–3.37	<0.001	1.65	1.20–2.29	0.002
APACHE2	1.05	1.03–1.07	<0.001	1.05	1.03–1.07	<0.001
**Model #2**						
lactate/albumin >0.15	2.5	1.85-3.37	<0.001	1.44	1.03–2.00	0.03
SAPS2	1.03	1.02–1.04	<0.001	1.03	1.02–1.04	<0.001
**Model #3**						
lactate/albumin >0.15	2.5	1.85–3.37	<0.001	2.94	2.39–3.60	<0.001
glucose (mmol/L)				1.01	0.99–1.03	0.41
leucocytes (G/L)				1.003	0.993–1.013	0.55
heart rate (bpm)				1.009	1.005–1.012	<0.001
ASAT (μmol/L·s)				1.004	0.995–1.013	0.41
ALAT (μmol/L·s)				1.00	0.980–1.019	0.97
BUN (mg/dL)				1.034	1.025–1.044	<0.001
creatinine (mg/dL)				0.999	0.998–0.999	<0.001
